# Assessing the knowledge, attitudes and barriers regarding health promotion of breast cancer among community pharmacists

**DOI:** 10.2144/fsoa-2022-0051

**Published:** 2023-02-03

**Authors:** Muna Oqal, Mohanad Odeh, Rawan Abudalu, Abdelrahim Alqudah, Roaa Alnajjar, Yazeed Bani Younes, Maram Al-Shawabkeh, Luma Taha

**Affiliations:** 1 Department of Pharmaceutics & Pharmaceutical Technology, Faculty of pharmaceutical sciences, The Hashemite University, PO Box 330127, Zarqa 13133, Jordan; 2 Department of clinical pharmacy & pharmacy practice, Faculty of pharmaceutical sciences, The Hashemite University, PO Box 330127, Zarqa 13133, Jordan

**Keywords:** attitudes, awareness, barriers, breast cancer, community pharmacists, educational material, health promotion, knowledge, patients, screening methods

## Abstract

**Aim:**

This study aimed to identify the perspective knowledge, attitudes, and barriers of community pharmacists in promoting breast cancer health.

**Methods:**

An internet-based self-administrated questionnaire was distributed using social media groups to the community pharmacists in Jordan.

**Results:**

A 76.7% of the pharmacists had insufficient knowledge score of breast cancer and 92.7% had positive attitude. Access to breast cancer educational materials was the major barrier to pharmacists. A significant association was found between pharmacists' knowledge and breast cancer educational materials being given to patients (p < 0.001).

**Conclusion:**

Despite the low breast cancer knowledge score and stated barriers that could prevent actualizing community pharmacists' role, they had positive attitude toward educating patients about breast cancer health.

## Plain language summary

The aim of this study was to evaluate pharmacists' knowledge, attitudes, and barriers about breast cancer health promotion among community pharmacists. An internet-based self-administrated questionnaire was distributed using social media groups to the community pharmacists in Jordan. A 76.7% of the pharmacists had poor knowledge score of breast cancer and 92.7% had positive attitude. The major barrier to pharmacists was access to breast cancer educational materials. A strong association was found between pharmacists' knowledge and breast cancer educational materials being given to patients. Also, community pharmacists had positive attitude toward educating patients about breast cancer health.

Cancer is a leading cause of death worldwide with more than 9.6 million deaths and 18.1 million new cases recorded in 2018. These figures are forecast to rise globally in an aging population in the coming two decades [1]. Cancer is a malignant disease which originates from a single cell that undergoes a multistep mechanism called carcinogenesis. This is where a single cell starts to behave abnormally, multiplies uncontrollably and persistently, accumulates changes, forming masses of cancerous cells called tumour, and eventually, invading neighboring tissues [2,3]. Note that breast cancer is the most predominant cancer and the leading cause of death among women in developing countries [4]. It has been estimated that 1 in every 200 women would develop breast cancer under the age of 40 years [5]. In addition, the International Agency for Research on Cancer estimates that there were 19.3 million new cancer cases and 10.0 million cancer deaths in 2020 [6]. In Jordan, the most common malignancy is breast cancer which is the third leading cause of cancer death after lung and colorectal cancers [7,8]. In addition, there is a significant increase in the number of new cases among younger women; who unfortunately present with more advance stage of breast cancer comparing to Western countries [7]. Similarly, many patients turn up to medical centers for the first time having advanced stages of breast cancer, even they received initiatives to screen for breast cancer. Avoidance of breast cancer screening could be due many reasons as lack of awareness of the importance of screening and partly probably, embarrassment of the screening methods [9]. This highlights the importance of increasing awareness of early screening for breast cancer among women and emphasizes on the chances of full recovery of early discovery of breast cancer which might improve survival rate [10–13]. Early detection of breast cancer includes both breast self-examination and clinical breast examination, which was recommended internationally including guidelines of the those by the American Cancer Society [14]. After detection of breast cancer, patients undertake long-term treatment modalities which require appropriate counseling and to adhering strictly to prescribed regimen; otherwise many of these treatment modalities are likely to fail [15].

One of the most accessible and trusted healthcare providers are pharmacists, who provide direct patient care centered on patient needs [16]. Pharmacists are expert in medications; providing patients with proper counseling and education on how to get the best benefit of their treatment modalities [17]. Several researches reported that counseling patients of breast cancer about chemotherapy and its associated side effects reduced side effects and improved the quality of life [18–20]. Moreover, counseling and educating patients of breast cancer about their medications led to decrease anxiety level and enhance the psychological outcomes in those patients in comparison to patients with inadequate counseling and education [21].

Noteworthy, pharmacists should be aware about chemotherapy prescribing errors and its tragic consequences for the oncology patients which lead to treatment failure in cancer patients in different protocols. It was reported in a cross-sectional observational study performed on 500 cancer patients, to identify the incidence of prescribing medication errors (PME) involving chemotherapeutic agents. Findings showed that all the cases contained at least one error and the risk factors predicting the prescribing errors were the protocol type, the tumor type, the toxicity type of the antineoplastic regimen which should be prevented for improvement of treatment plan [22]. In addition, pharmacists play a vital role in decreasing health literacy of community. A study was conducted in an emergency hospital on 1025 and 1024 patients to detect the rate and the severity of medication errors using direct observation before and after the implementation of educational tools. The findings of this study highlight the importance of pharmacist interventions in declining all types of medication errors and in significantly reducing the rate of medication errors in both pre-intervention and post-intervention phase. Noteworthy, clinical pharmacist interventions which improved the knowledge and awareness of nurses about medication errors in an emergency hospital environment were shown to be effective in reduction of the rate and severity of medication administration errors among nurses [23].

Community pharmacies are easily accessible to patients due to their location which is widely distributed within the communities, in addition to the extended working h, and free counseling about medications being dispensed in the pharmacy [24]. Therefore, community pharmacists can play an integral role in increasing the awareness of breast cancer especially in women who visit community pharmacies considerably [25,26]. Pharmacists have considerable knowledge of topics related to breast cancer and presumably can play an important role in supporting breast cancer health promotion among patients [16,27,28]. Notably, many studies have been conducted to assess the pharmacists' understanding about breast cancer health promotion and found a significant gaps in their knowledge [29–31].

A search of the literature revealed one study has been conducted in Jordan to assess the knowledge, attitudes, and barriers of community pharmacists toward breast cancer health promotion since 2015 [32]. Up to my knowledge, no studies have been set out to explore further gaps, improvements, and subtle changes over time of the knowledge, attitudes, and barriers of community pharmacists concerning health promotion of breast cancer in in Jordan especially after COVID-19 pandemic, and their role in promoting the health of patients with breast cancer visiting the pharmacies. In addition, this study aimed at identifying predictors of the knowledge in association with community pharmacists' sociodemographic and practice variables of their knowledge and attitudes toward breast cancer health promotion.

## Methods

### Design & data collection

To meet the study objectives a cross-sectional design was carried out. Data were collected between the period January–March 2022 using an internet-based self-administrated questionnaire which was created using Google Forms. The participants in our study were recruited through social media platforms. The questionnaire was distributed across several Facebook and WhatsApp groups of pharmacists among different areas in Jordan. These social media groups were created as a tool for general communication within the pharmacist's community. Informed consent was obtained from the participants as a pre-request to proceed in participation.

### Sample size

The sample size was calculated based on 95% confidence level, and 5% confidence interval, and total pharmacists in Jordan is 20,000. The sample size calculation revealed the need for at least 378 pharmacists. However, for the purpose of enhancing the generalizability of the results, a minimum sample of 605 pharmacists was intended.

### Study tool

**Figure 1.**
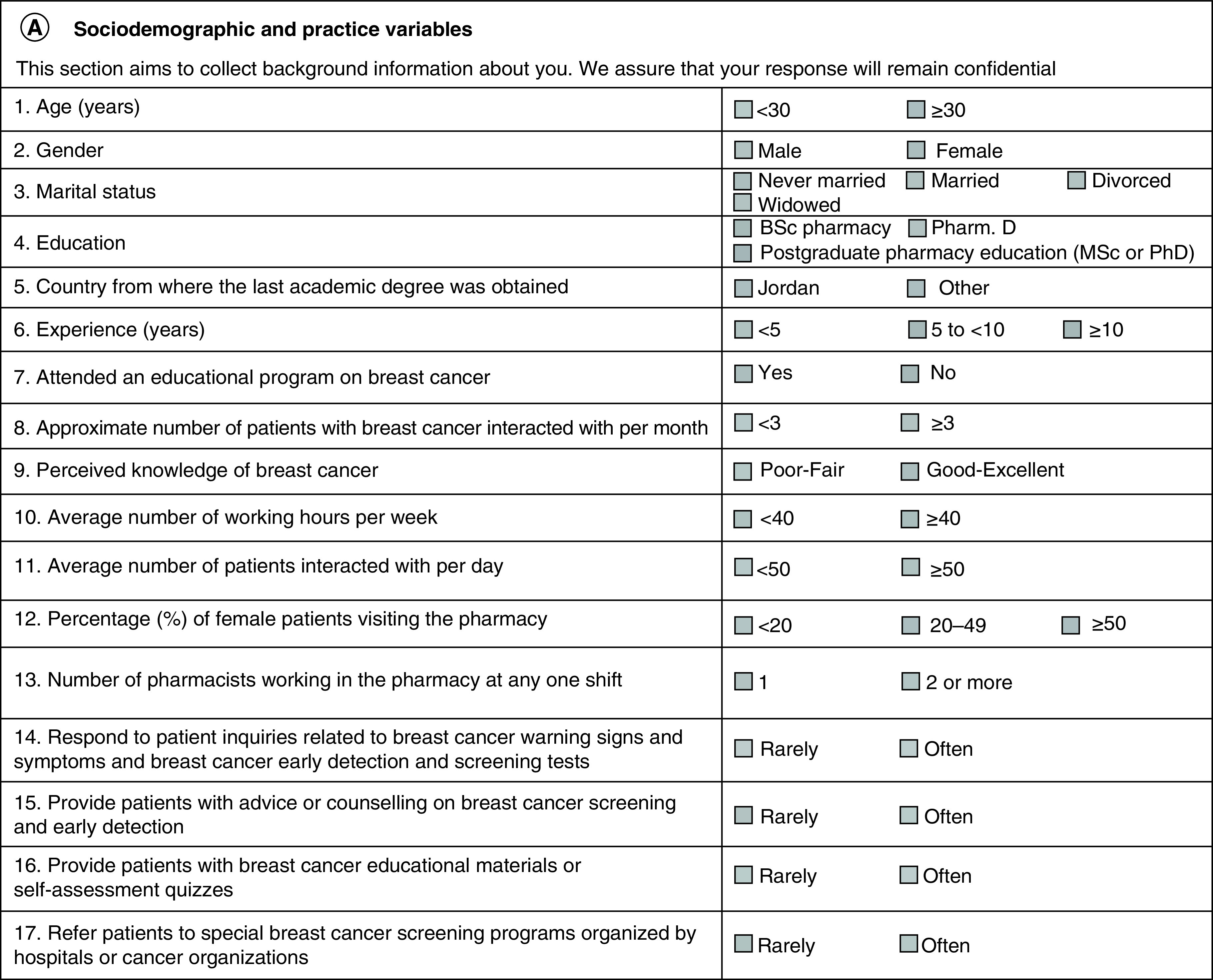

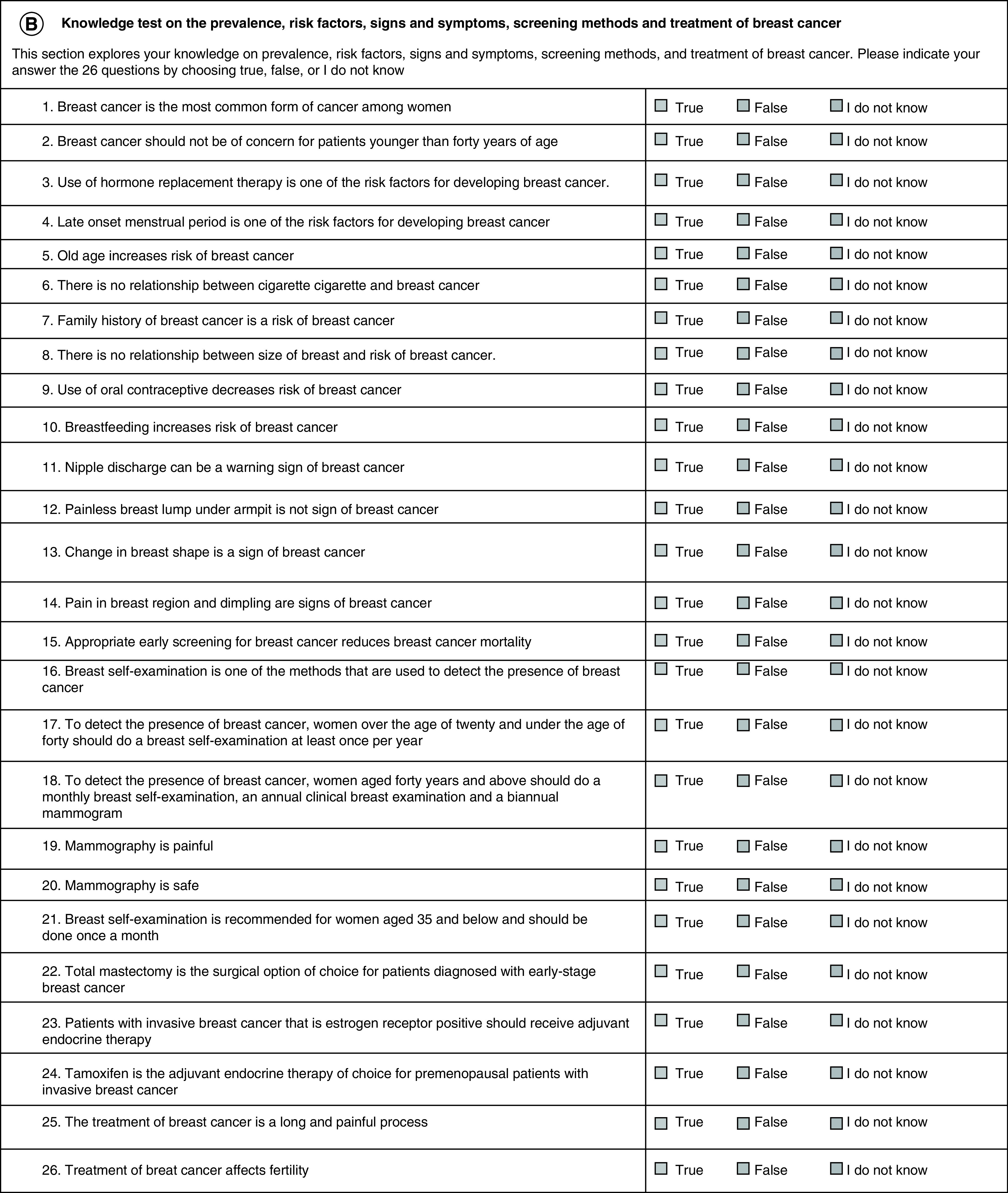

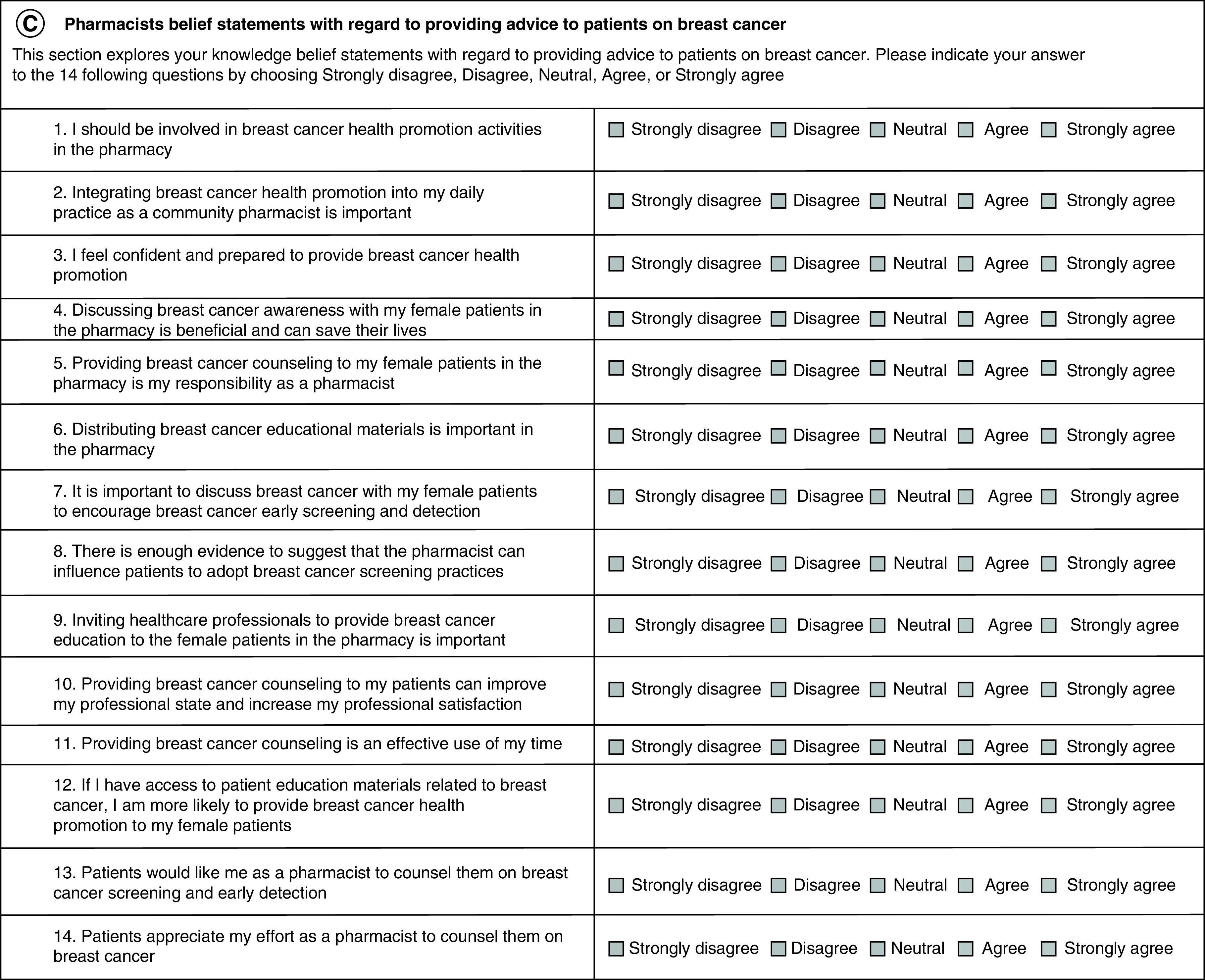

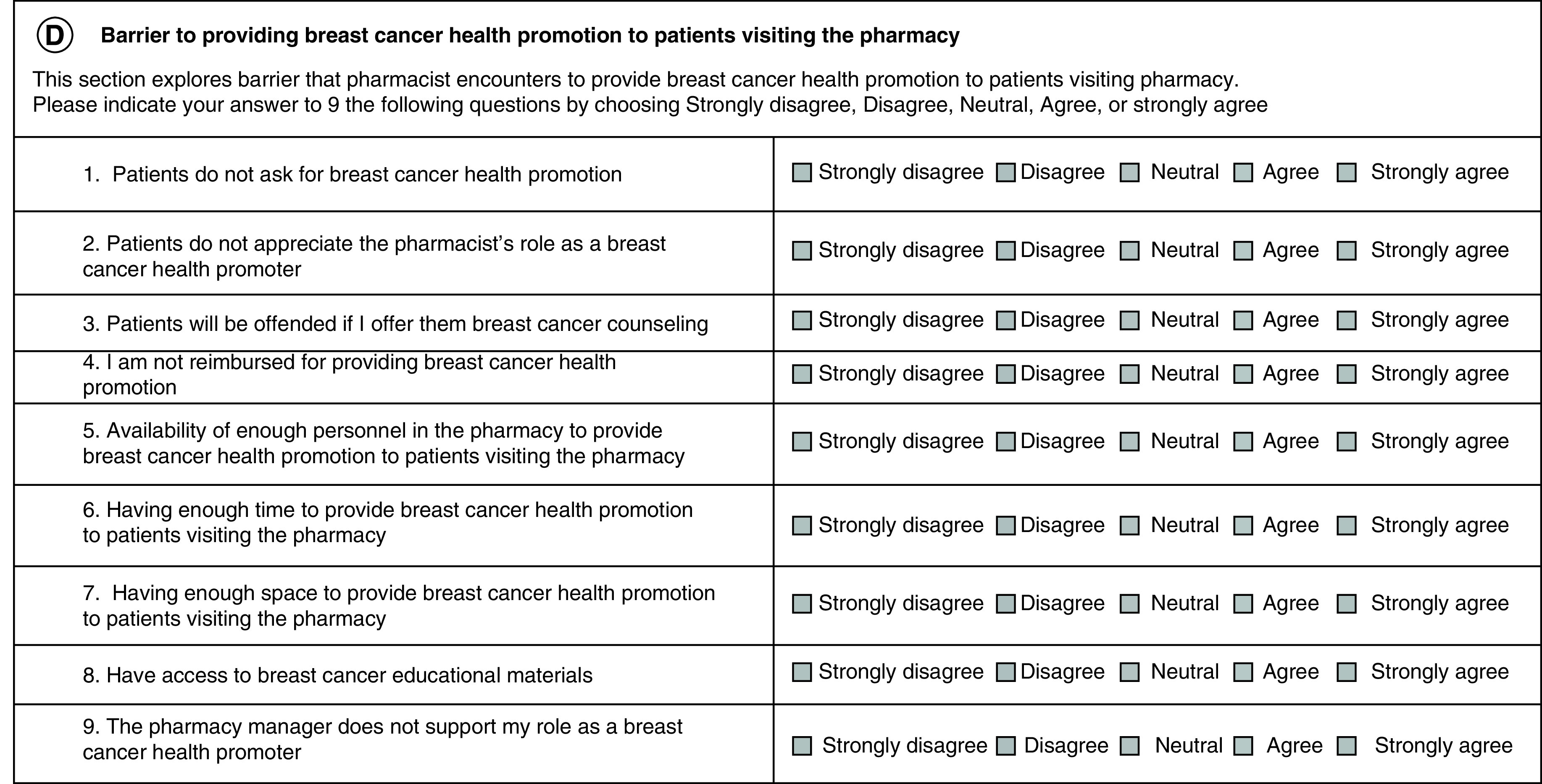
A detailed questionnaire of this study. **(A)** Sociodemographic and practice variables. **(B)** Knowledge test on the prevalence, risk factors, signs and symptoms, screening methods and treatment of breast cancer. **(C)** Pharmacists belief statements with regard to providing advice to patients on breast cancer. **(D)** Barrier to providing breast cancer health promotion to patients visiting the pharmacy.

The questionnaire was created especially for the purpose of this study. Most of the questions related to the knowledge, attitudes, and barriers were selected from the literature [32–34]. As was outlined in a previous study published in 2021 [34], the questionnaire was divided in to 4 sections – the first section included the sociodemographic and practice variables of community pharmacists, the second section contained 26 items to test the knowledge of the community pharmacists about breast cancer prevalence, risk factors, signs and symptoms, screening methods, and treatment, the third section used 14 questions to study the attitudes and beliefs of the pharmacists and the final section included 9 questions related to the barriers faced by pharmacists in promoting breast cancer health to patients coming into their pharmacy. The detailed questionnaire can be seen in Figure 1.

### Face validity & pilot testing

Face validity was revised by a group of experts in the field and was constituted of two oncologists, two community pharmacists, and two clinical pharmacists. A scale of 1–5 (1 indicated not relevant and 5 indicated highly relevant) was used to rate each item in the questionnaire. Items included in the final questionnaire were rated as relevant and highly relevant by the reviewers. Conflicting ratings were resolved by discussion and consensus. To ensure comprehensibility and clarity of the questionnaire, pilot test was performed. 20 community pharmacists were asked to answer the questionnaire, then after a short period (one week), the same pharmacists were answered the questionnaire in a second round. In addition, the stability and reliability of the questionnaire was confirmed in another study [31], where the stability of scores over a short period of time was accepted as Pearson's correlation coefficients: 90%, 95% CI: 0.91–0.99, p-value < 0.001. The questionnaire internally consistent was also accepted, Cronbach's alpha: 83.9%, 95% CI: 81.4–85.9%, p-value < 0.001.

### Data analysis & statistical method

Data collected were analyzed using the Statistical Package for Social Sciences version 25.0 (SPSS). Categorical and ordinal variables were shown as frequencies (n) and percentages (%). Univariate statistical analysis was used to determine whether there was an association between the variables, the χ^2^ test for association was used. Phi (Φ) factor (or Cramer's V coefficient, when having more than two dichotomous variables) was used to evaluate the strength of association of a nominal-by-nominal relationship (and is a measure of effect size), where the value of 1 indicates a complete association, “0” indicating no association, small association 0.1, medium association 0.3 and large association 0.5. Phi (Φ) or Cramer's V coefficient was only illustrated when there is a statistical significance. Multivariable logistic regression (backward stepwise) model was used to eliminate independent predictors with non-statistically significant contributions (i.e., P more than 0.05). For evaluating the quality of the logistic regression results (i.e., model fit), the overall statistical significance tests for models were carried out. The Omnibus Test of Model Coefficients was used to measure how well the final model predicted categories compared with the baseline model and other previous models in the backward stepwise approach (i.e., p for the final model should be < 0.05). To understand how much variation in the dependent variable can be explained by the model; the Nagelkerke R Square (R^2^) was reported [35,36]. To assess the effectiveness of the predicted classification against the actual classification, percentage accuracy in classification, sensitivity and specificity were calculated and reported. To align with the standard recommendations for logistic model output quality, discriminative ability testing was carried out through Receiver Operating Characteristic (ROC) curve analysis [36,37]. ROC results were used to evaluate the ability of the final models to identify the predicted readmission status compared with the observed actual readmission status. Area Under the Curve (AUC) represents the overall accuracy of logistic prediction models (sensitivity and specificity matrix), in which AUC = 1.00 means 100% accurate [38,39].

### Method for scoring

#### Knowledge

Each question with a correct answer, a 1 point, and a wrong answer -0.5 point. Answer with did not know = 0.0 point. As the present study has 26 questions, the highest possible score = 26 points and the minimum possible score = -13 points. Participants were categorized into binary classification based on their score (<50%, or >50%).

#### Attitude & barrier

The following points were assigned to answer questions: strongly disagree (-2), disagree (-1), neutral (0), agree (1), strongly agree (2).

Attitude has 14 statements, accordingly, the highest possible score = 28 points, the minimum possible score = -28 points. Based on attitude total points the participants were categorized into negative, neutral and positive attitude groups, with a total point from -28 to -1, zero and from +1 to +28, respectively.

Perceived Barriers were 9 statements accordingly, the highest possible score = 18, the minimum possible score = -18. Based on Barriers total points the participants were categorized into negative, barrier dominant neutral, and not barrier dominant groups, with a total point from -18 to -1, 0, and from +1 to +18, respectively.

## Results

### Demographic information for the responders

A total of 605 pharmacists successfully participated in the present study (response rate: 90.3%). As expected, females (56.2%) pharmacists were more than males (43.8%). Pharmacists younger than 30 years (54%) were more than those older than 30 (46%). Most responders were having BSc Pharmacy (Almost 69%), almost one-quarter of pharmacists (24%) were PharmDs and less than 10% were postgraduates. Pharmacists with less than 5 years' experience were 42.1%, and those with more than 10 years' experience were 20%. More than half of pharmacists had attended educational programs (Almost 60%), perceived their knowledge about breast cancer as good–excellent (65.1%), worked more than 40 h per week (59.5%), worked in a pharmacy with 2 or more pharmacists (61.3%), answered with the often option for the following statements: response to breast cancer warning signs and symptoms and breast cancer early detection and screening tests (64.1%), provide patients with breast cancer educational materials or self-assessment quizzes (55%) and refer patients to special breast cancer screening programs organized by hospitals or cancer organizations (54.2%). All demographic variables are listed in Table 1.

**Table 1.** Sociodemographic and practice variables of the pharmacists who took part in the study (n = 605).

Variable	n	%
Age (years)		
<30	327	54.0
≥30	278	46.0
Sex		
Male	265	43.8
Female	340	56.2
Marital status		
Never married	273	45.1
Married/divorced/widowed	332	54.9
Education		
BSc Pharmacy	415	68.6
Pharm.D	147	24.3
Postgraduate pharmacy education (MSc or PhD)	43	7.1
Place from where the academic degree was obtained		
Jordan	526	86.9
Other	79	13.1
Experience (years)		
<5	255	42.1
5 to <10	229	37.9
≥10	121	20.0
Attended an educational program on breast cancer		
Yes	360	59.5
No	245	40.5
Approximate patients with breast cancer interacted with per month (n)		
<3	382	63.1
≥3	223	36.9
Perceived knowledge of breast cancer		
Poor–Fair	211	34.9
Good–Excellent	394	65.1
Average working h per week (n)		
<40	245	40.5
≥40	360	59.5
Average patients interacted with per day (n)		
<50	272	45.0
≥50	333	55.0
Female patients visiting the pharmacy (%)		
<20	81	13.4
20–49%	302	49.9
≥50	222	36.7
Pharmacists working in the pharmacy at any one shift (n)		
1	234	38.7
2 or more	371	61.3
Respond to patient inquiries related to breast cancer warning signs and symptoms and breast cancer early detection and screening tests		
Rarely	217	35.9
Often	388	64.1
Provide patients with advice or counseling on breast cancer screening and early detection		
Rarely	208	34.4
Often	397	65.6
Provide patients with breast cancer educational materials or self-assessment quizzes		
Rarely	272	45.0
Often	333	55.0
Refer patients to special breast cancer screening programs organized by hospitals or cancer organizations		
Rarely	277	45.8
Often	328	54.2

### Scores & categories for knowledge, attitude & barrier

Table 2 illustrates the scores and categories for knowledge, attitude and barrier. As seen in table, the majority of responders (76.7%) have a knowledge score less than 50% with mean 8.2 (2.9) out of 26. Positive attitude was noticed for almost 93% of responders with a mean 17.46 (5.9) out of 28. The percentage of responders who perceived barrier versus perceived no barriers were almost closed to each other (41.5% vs 48.1%).

**Table 2.** The groups and categories for knowledge, attitude and barrier points.

Group	Categories	n (%)	Mean (standard deviation)	Median (interquartile range)	Minimum	Maximum
Knowledge	Less than 50%	464 (76.7)	8.2 (2.9)	8.5 (6–10)	-1.5	12.5
	More than or equal 50%	141(23.3)	15.3 (1.6)	15.5 (14–16)	13	20.5
	Total	605	9.8 (4.1)	9.5 (7–12.5)	-1.5	20.5
Attitude	Negative	36 (6%)	-11.7 (8.5)	-11.5 (-16.5 to -3.5)	-28	-1
	Neutral	8 (1.3%)	00	00	00	00
	Positive	561 (29.7%)	17.46 (5.9)	17.0 (14–19)	1	28
	Total	605 (100%)	15.49 (9.4)	17.0 (13.0–21.0))	-28	28
Barrier	Barrier Dominant	251 (41.5%)	5.7 (4.3)	4 (2.0–9.0)	1	18
	Neutral	63 (10.4%)	0	0	0	0
	Not Barrier Dominant	291 (48.1%)	-5.0 (3.1)	-5.0 (-7.0 to -2.0)	-18	-1
	Total	605 (100.0%)	-0.15 (6.1)	0.0 (-5.0 to 3.0)	-18	18

### Results for knowledge

Despite that 65.1% claimed perceived good–excellent knowledge about breast cancer, only 23.1% (n: 141) had a knowledge score equal to or more than 50%. However, the vast majority of pharmacists correctly identified breast cancer as the most common cancer among females (87.4%). As seen in Table 3, there were obvious variances among each knowledge sector (prevalence, risk factors, signs and symptoms, screening methods and treatment). The best-reported knowledge domain was the sign and symptoms, where the majority of pharmacists had identified the correct signs and symptoms for 3 out of 4 statements in that domain; nipple discharge (75.9%), change in breast shape (84%), and pain in breast region and dimpling (77%). This was followed by correctly identifying risk factors such as using hormone replacement therapy (77%), being old age (81.7%), and having a family history (82.1%). It was noticed when the correct answer was (false) most pharmacists failed to identify it, for example only 7.9% identify the correct answer for the statement (to detect the presence of breast cancer, women aged 40 years and above should do a monthly breast self-examination, an annual clinical breast examination, and a biannual mammogram), only 12.6% identify the correct answer for (to detect the presence of breast cancer, women over the age of twenty and under the age of forty should do a breast self-examination at least once per year), and only 14.5% get the correct answer for statement (late-onset menstrual period is one of the risk factors for developing breast cancer).

**Table 3.** Responses of the community pharmacists on the 26-item knowledge test on the prevalence, risk factors, signs and symptoms, screening methods, and treatment of breast cancer.

Question	Standard answer (true, false)	Correct answer		Incorrect answer		Did not know	
		n	%	n	%	n	%
Prevalence of breast cancer							
Breast cancer is the most common form of cancer among women.	T	529	87.4	52	8.6	24	4.0
Breast cancer should not be of concern for patients younger than forty years of age.	F	235	38.8	337	55.7	33	5.5
Risk factors							
Use of hormone replacement therapy is one of the risk factors for developing breast cancer.	T	466	77.0	76	12.6	63	10.4
Late onset menstrual period is one of the risk factors for developing breast cancer.	F	88	14.5	425	70.2	92	15.2
Old age increase risk of breast cancer.	T	494	81.7	69	11.4	42	6.9
There is no relationship between cigarette smoking and breast cancer.	F	253	41.8	305	50.4	47	7.8
Family history of breast cancer is a risk of breast cancer.	T	497	82.1	59	9.8	49	8.1
Use of oral contraceptive decrease risk of breast cancer.	F	196	32.4	326	53.9	83	13.7
There is no relationship between size of breast and risk of breast cancer.	F	117	19.3	401	66.3	87	14.4
Breastfeeding increase risk of breast cancer.	F	257	42.5	283	46.8	65	10.7
Signs and symptoms							
Nipple discharge can be a warning sign of breast cancer.	T	459	75.9	74	12.2	72	11.9
Painless breast lump under armpit is not sign of breast cancer.	F	226	37.4	316	52.2	63	10.4
Change in breast shape is a sign of breast cancer.	T	508	84.0	60	9.9	37	6.1
Pain in breast region and dimpling are signs of breast cancer.	T	466	77.0	88	14.5	51	8.4
Screening methods							
Appropriate early screening for breast cancer reduces breast cancer mortality.	T	493	81.5	72	11.9	40	6.6
Breast self-examination is one of the methods that are used to detect the presence of breast cancer.	T	494	81.7	65	10.7	46	7.6
To detect the presence of breast cancer, women over the age of twenty and under the age of forty should do a breast self-examination at least once per year.	F	76	12.6	485	80.2	44	7.3
To detect the presence of breast cancer, women aged forty years and above should do a monthly breast self-examination, an annual clinical breast examination and a biannual mammogram.	F	48	7.9	507	83.8	50	8.3
Mammography is painful.	F	183	30.2	303	50.1	119	19.7
Mammography is safe.	T	415	68.6	92	15.2	98	16.2
Breast self-examination is recommended for women aged 35 and below and should be done once a month.	T	465	76.9	91	15	49	8.1
Treatment							
Total mastectomy is the surgical option of choice for patients diagnosed with early-stage breast cancer.	F	167	27.6	353	58.3	85	14
Patients with invasive breast cancer that is estrogen receptor positive should receive adjuvant endocrine therapy.	T	416	68.8	72	11.9	117	19.3
Tamoxifen is the adjuvant endocrine therapy of choice for premenopausal patients with invasive breast cancer.	T	465	76.9	47	7.8	93	15.4
The treatment of breast cancer is a long and painful process.	T	403	66.6	115	19	87	14.4
Treatment of breast cancer affects fertility.	F	126	20.8	373	61.7	106	17.5

### Results for attitude

There was a common notice that a positive attitude is more than a negative. Across all questions, more than 70% of the pharmacists responded to each question with agreeing or strongly agreeing. This resulted in a positive attitude score for 92.7% of the participated pharmacists. Amazingly, the positive attitude of pharmacists was not associated with the level of knowledge, in which the attitude score demonstrated no association with the knowledge score (χ^2^ [3.8]; p = 0.15). As seen in Table 4, the vast majority (84.6%) of the pharmacists agreed that they should be involved in breast cancer health promotion activities in the pharmacy, 82.1% agreed that the distribution of breast cancer educational materials is important in the pharmacy, 81.7% agreed that discussing breast cancer awareness is beneficial and can save female's lives.

**Table 4.** Responses of the pharmacists on the belief statements with regard to providing advice to patients on breast cancer.

Statements	Sum agree	%	Neutral	%	Sum disagree	%
I should be involved in breast cancer health promotion activities in the pharmacy	512.0	84.6%	59	9.8%	34	5.6%
Integrating breast cancer health promotion into my daily practice as a community pharmacist is important	473.0	78.2%	96	15.9%	36	6.0%
I feel confident and prepared to provide breast cancer health promotion	429.0	70.9%	124	20.5%	52	8.6%
Discussing breast cancer awareness with my female patients in the pharmacy is beneficial and can save their lives	494.0	81.7%	85	14.0%	26	4.3%
Providing counseling of dispensed medications to breast cancer female patients in the pharmacy is my responsibility as a pharmacist	470.0	77.7%	99	16.4%	36	6.0%
Distributing breast cancer educational materials is important in the pharmacy	497.0	82.1%	84	13.9%	24	4.0%
It is important to discuss breast cancer with my female patients to encourage breast cancer early screening and detection	487.0	80.5%	80	13.2%	38	6.3%
There is enough evidence to suggest that the pharmacist can influence patients to adopt breast cancer screening practices	468.0	77.4%	97	16.0%	40	6.6%
Inviting healthcare professionals to provide breast cancer education to the female patients in the pharmacy is important	461.0	76.2%	106	17.5%	38	6.3%
Providing breast cancer counseling to my patients can improve my professional state and increase my professional satisfaction	491.0	81.2%	78	12.9%	36	6.0%
Providing breast cancer counseling is an effective use of my time	485.0	80.2%	84	13.9%	36	6.0%
If I have access to patient education materials related to breast cancer, I am more likely to provide breast cancer health promotion to my female patients	481.0	79.5%	93	15.4%	31	5.1%
Patients would like me as a pharmacist to counsel them on breast cancer screening and early detection	465.0	76.9%	103	17.0%	37	6.1%
Patients appreciate my effort as a pharmacist to counsel them on breast cancer	457.0	75.5%	118	19.5%	30	5.0%

The weakest attitude was noted in response to the statement “I feel confident and prepared to provide breast cancer health promotion”. However, even for this question particularly, the specific attitude for answering the question was not associated with the score of participant knowledge (χ^2^(4) = 2.9; p = 0.57).

### Results for barriers

Unlike attitude, pharmacists perceived barriers in various ways. The agreement on barriers was ranged from 19.2% to 56.7%. As seen in Table 5, the strongest barrier as perceived by pharmacists was having access to breast cancer educational materials (agreed by 56.7%), followed by having enough time to provide breast cancer health promotion to patients visiting the pharmacy and having enough space to provide breast cancer health promotion to patients visiting the pharmacy (agreed by 51.4% and 51.2%, respectively). Amazingly, only 19.2% agreed that patients do not appreciate the pharmacist's role as a breast cancer health promoter, which means that patients' appreciation is not a barrier. In a similar context, pharmacists' reimbursement and patients' offense were not perceived as barriers, i.e., only 23% and 23.5% of pharmacists consider reimbursement and offended patients as barriers.

**Table 5.** Barrier to providing breast cancer health promotion to patients visiting the pharmacy.

Statements	Sum agree	%	Neutral	%	Sum disagree	%
Patients do not ask for breast cancer health promotion	172	28.4%	146	24.1%	287	47.4%
Patients do not appreciate the pharmacist's role as a breast cancer health promoter	116	19.2%	147	24.3%	339	56.0%
Patients will be offended if I offer them breast cancer counseling	142	23.5%	158	26.1%	305	50.4%
I am not reimbursed for providing breast cancer health promotion	139	23.0%	197	32.6%	269	44.5%
Availability of enough personnel in the pharmacy to provide breast cancer health promotion to patients visiting the pharmacy	227	37.5%	196	32.4%	182	30.1%
Having enough time to provide breast cancer health promotion to patients visiting the pharmacy	311	51.4%	136	22.5%	158	26.1%
Having enough space to provide breast cancer health promotion to patients visiting the pharmacy	310	51.2%	164	27.1%	131	21.7%
Have access to breast cancer educational materials	343	56.7%	156	25.8%	106	17.5%
The pharmacy manager does not support my role as a breast cancer health promoter	215	35.5%	159	26.3%	231	38.2%

A sum-up of barrier score revealed that 48.1% (i.e., barrier score was negative) of pharmacists do not have a strong barrier to delivering breast cancer care, while 41.5% (i.e., barrier score was positive) of pharmacists claim barrier that prevents them to deliver the service. 10.4% would have a balance status (i.e., barrier score was zero). Contingency table analysis showed that pharmacists with a knowledge score of more than 50% perceived higher levels of total barrier score compared with pharmacists who had a knowledge score of less than 50% (55.3% vs 37.3%, p < 0.001). The more knowledge the more realization of the barriers. Analysis showed also that perceived barriers had no association with attitude (p = 0.6), despite perceived barriers most pharmacists (92.7%) have a positive attitude toward delivering services. Attitudes did not impact the perceived barriers.

### Univariate analysis for association with Knowledge score

Table 6 shows that most demographic factors have a statistically significant association with the knowledge score. Accordingly, strength of association was used to get a better understanding of the association, and more impactful a stepwise backward multivariable logistic regression was carried out. The strongest and statistically significant association was noticed between knowledge score and the number of pharmacists in the pharmacy, i.e., having 1 pharmacist scored a higher percentage of knowledge score (>50%) compared with pharmacy who had 2 or more pharmacists (41.5% vs 11.69%; p < 0.0001; Phi: 0.34). Most variables at the univariate level of analysis demonstrated a statistically significant association, so multivariable analysis was carried out for better interpretation. Amazingly, the perceived knowledge filled by pharmacists -i.e., self-evaluation was not associated with the knowledge score (p = 0.17), and the educational level was not associated with the knowledge score (p = 0.066).

**Table 6.** Associations between sociodemographic and practice variables of the pharmacists and scoring 50?% or more in the knowledge test.

Knowledge score									
Variable	n	%	<50%		≥50%		χ^2^ Test	p-value	Association strength
			n = 464	%	n = 141	%			
Age (years)									
<30	327	54.0	240	73.4%	87	26.6%	4.335^a^	0.037	0.085
≥30	278	46.0	224	80.6%	54	19.4%			
Sex									
Female	340	56.2	244	71.8%	96	28.2%	10.553^a^	0.001	0.13
Male	265	43.8	220	83.0%	45	17.0%			
Marital status									
Never married	273	45.1	196	71.8%	77	28.2%	6.7	0.009	0.07
Married/divorced/widowed	332	54.9	268	80.7%	64	19.3%			
Education									
BSc Pharmacy	415	68.6	308	74.2%	107	25.8%	5.431^a^	0.066	
Pharm.D	147	24.3	123	83.7%	24	16.3%			
Postgraduate pharmacy education (MSc or PhD)	43	7.1	33	76.7%	10	23.3%			
Place from where the academic degree was obtained									
Jordan	526	86.9	391	74.3%	135	25.7%	12.548^a^	<0.001	-0.14
Other	79	13.1	73	92.4%	6	7.6%			
Experience (years)									
<5	255	42.1	167	65.5%	88	34.5%	31.912^a^	<0.001	0.23
5 to <10	229	37.9	198	86.5%	31	13.5%			
≥10	121	20.0	99	81.8%	22	18.2%			
Attended an educational program on breast cancer									
Yes	360	59.5	294	81.7%	66	18.3%	12.297^a^	<0.001	0.14
No	245	40.5	170	69.4%	75	30.6%			
Approximate patients with breast cancer interacted with per month (n)									
<3	382	63.1	275	72.0%	107	28.0%	12.834^a^	0.000	0.15
≥3	223	36.9	189	84.8%	34	15.2%			
Perceived knowledge of breast cancer									
Poor–Fair	211	34.9	155	73.5%	56	26.5%	1.896^a^	0.168	0.06
Good–Excellent	394	65.1	309	78.4%	85	21.6%			
Average working h per week (n)									
<40	245	40.5	178	72.7%	67	27.3%	3.762^a^	0.052	0.08
≥40	360	59.5	286	79.4%	74	20.6%			
Average patients interacted with per day (n)									
<50	272	45.0	193	71.0%	79	29.0%	9.104^a^	0.003	0.12
≥50	333	55.0	271	81.4%	62	18.6%			
Female patients visiting the pharmacy (%)									
<20	81	13.4	62	76.5%	19	23.5%	24.535^a^	<0.001	0.2
20–49%	302	49.9	208	68.9%	94	31.1%			
≥50	222	36.7	194	87.4%	28	12.6%			
Pharmacists working in the pharmacy at any one shift (n)									
1	234	38.7	137	58.5%	97	41.5%	70.306^a^	<0.001	0.34
2 or more	371	61.3	327	88.1%	44	11.9%			
Respond to patient inquiries related to breast cancer warning signs and symptoms and breast cancer early detection and screening tests									
Rarely	217	35.9	163	75.1%	54	24.9%	0.472^a^	0.492	
Often	388	64.1	301	77.6%	87	22.4%			
Provide patients with advice or counseling on breast cancer screening and early detection									
Rarely	208	34.4	159	76.4%	49	23.6%	0.011^a^	0.916	
Often	397	65.6	305	76.8%	92	23.2%			
Provide patients with breast cancer educational materials or self-assessment quizzes									
Rarely	272	45.0	176	64.7%	96	35.3%	39.735^a^	<0.001	0.26
Often	333	55.0	288	86.5%	45	13.5%			
Refer patients to special breast cancer screening programs organized by hospitals or cancer organizations									
Rarely	277	45.8	194	70.0%	83	30.0%	13.228^a^	<0.001	0.15
Often	328	54.2	271	82.6%	57	17.4%			

### Multivariate analysis for association with knowledge score

**Figure 2.**
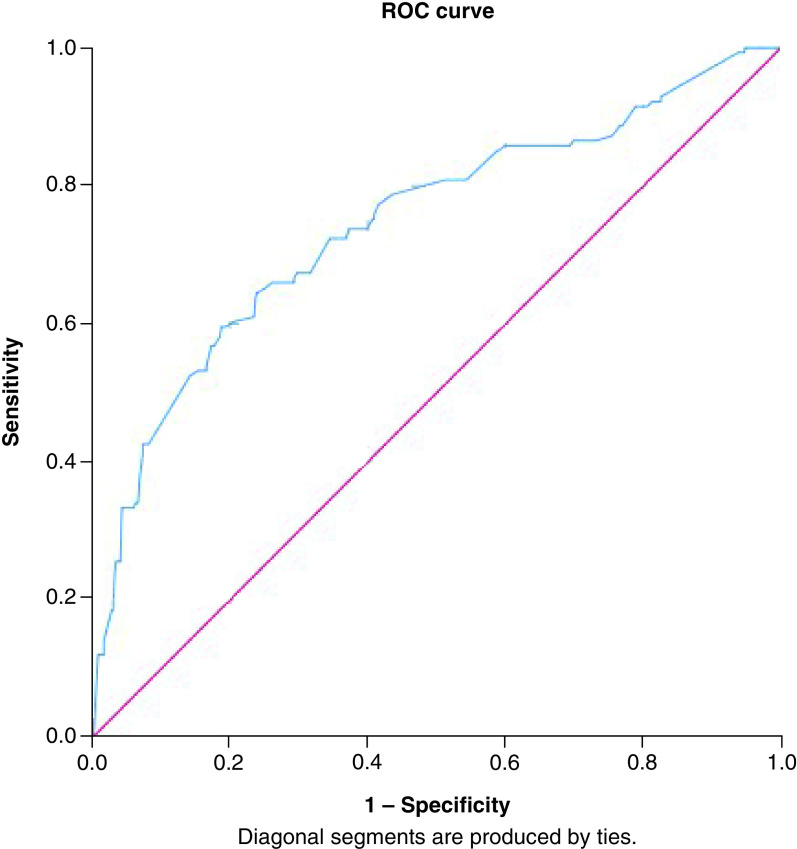
The receiver operating characteristic curve analysis, Area under the curve for multivariate regression analysis of the association between sociodemographic and practice variables with scoring 50% or more in the knowledge test. ROC: Receiver operating characteristic.

Binomial logistic regression (backward stepwise) was used to eliminate independent predictors with a non-statistically significant contribution. The best fit binomial logistic regression (backward stepwise) was a 10 steps model. The logistic regression model was statistically significant, χ^2^ (13) = 300.5, p < 0.0005. The model explained 52.0% (Nagelkerke R^2^) of the variance in knowledge score and correctly classified 80.0% of cases. Sensitivity was 35.7%, and specificity was 94.4%. The area under the ROC curve demonstrated acceptable discrimination of 74.5% (95% CI: 0.69 to 0.79, p < 0.0005), see Figure 2.

Only variables in Table 7 were the statistically significant variables to predict the knowledge score. The following variables were considered for the first step entry: variable(s) entered on step 1: age (years), gender, marital status, education, country from where your last academic degree was obtained, experience (years), attended an educational program on breast cancer, approximate number of patients with breast cancer interacted with per month, perceived knowledge of breast cancer, average number of working h per week, average number of patients interacted with per day, percentage (%) of female patients visiting the pharmacy, number of pharmacists working in the pharmacy at any one shift [34]. In addition to the answer the following statements (rarely or often): Respond to patient inquiries related to breast cancer warning signs and symptoms and breast cancer early detection and screening tests, provide patients with advice or counseling on breast cancer screening and early detection, provide patients with breast cancer educational materials or self-assessment quizzes, refer patients to special breast cancer screening programs organized by hospitals or cancer organizations. As seen in Table 7, the odds of having knowledge score >50% is 3.8 (p > 0.0001) times greater for a single pharmacist working in the pharmacy at any one shift opposed to more than two pharmacies. (OR: 3.812; 95% CI: 2.35–6.19).

**Table 7.** Multivariate regression analysis of the association between sociodemographic and practice variables with scoring 50?% or more in the knowledge test.

Variables in the equation	Subcategories	β	SE	Wald	Sig.	ODDs Ratio	95% CI for odds ratio	
							Lower	Upper
Education	Postgraduate education	Reference		5.456	0.065			
	BSc Pharmacy	-0.712	0.351	4.087	0.043	0.492	0.247	0.979
	Pharm.D	-0.921	0.407	5.159	0.023	0.396	0.178	0.881
Experience (years)	≥10	Reference		13.694	**0.001** ^†^			
	<5	0.372	0.308	1.460	0.227	1.451	0.793	2.655
	5 to <10	-0.690	0.323	4.560	0.033	0.501	0.266	0.945
Female patients visiting the pharmacy (%)	≥50	Reference		4.849	0.089			
	<20	-0.276	0.365	0.569	0.450	0.759	0.371	1.553
	20%–49%	0.358	0.258	1.933	0.164	1.431	0.864	2.370
Pharmacists working in the pharmacy at any one shift (n)	2 or more	Reference						
	1	1.338	0.247	29.353	**0.000** ^†^	3.812	2.349	6.186
Attended an educational program on breast cancer	No	Reference						
	Yes	-0.504	0.214	5.528	**0.019** ^†^	0.604	0.397	0.920
Provide patients with advice or counseling on breast cancer screening and early detection	Rarely	Reference						
	Often	-1.187	0.263	20.349	**0.000** ^†^	0.305	0.182	0.511
Provide patients with breast cancer educational materials or self-assessment quizzes	Rarely	Reference						
	Often	0.831	0.250	11.031	**0.001** ^†^	2.295	1.406	3.746

† Statistically significant predictors.

Pharmacists who often provide patients with breast cancer educational materials or self-assessment quizzes would be 2.3 (p < 0.001) times more likely to have >50% knowledge score compared with those who rarely provide such service (OR: 2.295; 95% CI: 1.41–3.75). Amazingly, with reference to analysis, pharmacists claimed attended an educational program on breast cancer or providing patients with advice or counseling on breast cancer screening and early detection were less likely to get >50% knowledge score, as ORs were 0.604 (95% CI: 0.397–0.920) and 0.305 (95% CI: 0.182–0.511); respectively.

## Discussion

Breast cancer is the most frequently diagnosed cancer among females worldwide [40]. It represents the warning to females' health in Jordan [7]. Self-examination and early screening for breast cancer diagnosis is significant to reduce morbidity and enhances the survival [41]. Nevertheless, numerous studies from developing countries as well as from Jordan have revealed the low level of awareness regarding breast cancer and its screening performance [42–44]. This highlights the need to broad the engagement of healthcare providers in promoting the awareness of breast cancer and its screening among public. The earlier detection of the cancer allows for the better and more effective management, unlike the disease diagnosis in advanced stages [45]. Community pharmacists are accessible healthcare providers who spend long h daily in direct contact with population and are recently progressively involved in variety of healthcare protective services [46,47]. Accordingly, they have ideal position to be integrated in promoting breast cancer health and awareness among women in community [32]. Therefore, this study was conducted to evaluate the knowledge, attitudes, and barriers of pharmacists in Jordan toward breast cancer health promotion. In addition, to highlight the gaps in some areas related to community pharmacists' awareness and perception in regard to breast cancer.

The questionnaire in this study was revalidated using Cronbach's alpha that showed an excellent reliability before it was distributed [48]. The sampling strategy is tried to include representative participants of community pharmacists in Jordan with both genders, various ages, education levels attained, and years of experience. As well, the questionnaire asked about participants perceived knowledge about breast cancer and asked about their practice toward patient-focused breast cancer awareness and counselling of early detection. This sample diversity might add validity and rigor to the obtained results in the study.

The key findings of this study indicate that the majority of participants lack sufficient knowledge about breast cancer as 76.9% participants had knowledge score less than 50. These results are consistent with data obtained in previous study in Jordan [32]. In contrast, the overall knowledge score was higher among community pharmacists in Qatar (50%) [49] and Palestine (63%) [50]. However, the vast majority of pharmacists correctly identified breast cancer as the most common cancer among females (87.4%). This finding matches those observed in earlier study that was conducted before 7 years in Jordan [51]. Most of community pharmacists in this study lack sufficient breast cancer particularly risk factors. They have shown lack of sufficient knowledge about breast cancer risk factors, in which they have a higher emphasis on genetic are hereditable causes [52]. While less than 50% of participants recognized the modifiable risk factors as cigarette smoking [53] and oral contraceptives [54] as potential risks and contribute to inferior prognosis. These results are in agreement with those obtained by previous research [55]. Moreover, community pharmacists were knowledgeable of the benefits of early screening in reducing breast cancer prognosis. However, knowledge gaps were recognized with mammography procedure, specifically, the female's age needs to conduct breast self-examination and mammography as this has been reported previously [49]. Although continuous efforts executed to increase public females' awareness regarding breast cancer, the screening level stays low [56]. Previous studies reported that healthcare providers orientation and attitude are pivotal factors to increase public awareness for early breast cancer screening programs [57,58]. This proves the need to involve healthcare providers in increasing patient breast cancer awareness for self-examination and encourage screening [55,59]. It is worthy to mention that patients' decision/choice in self-care is also a contributing factor to improve health outcomes [60]. Thus, it is crucial to improve community pharmacists' knowledge with regard to breast cancer screening recommendations by increasing the continuous educational programs organised by professional associations and encourage them to attend these activities [27]. In addition, previous studies have reported the orientation and attitude of healthcare providers have a great influence on women's awareness for early detection of the disease by screening methods [61]. Meanwhile, adoption of the oncology courses in pharmacy curriculum of undergraduate students would improve the roles for graduated pharmacists in raising the awareness and engagement in the screening programs [62,63]. Knowledge gaps regarding breast cancer treatment modalities were indicated in this study (less than 50% of pharmacists identified the adverse effects of breast cancer) and as similar gaps was reported in previous studies in Qatar [49] and Palestine [50].

The overall knowledge score was significantly associated with participant's gender, age, years of experience, and the place from where the academic degree was gained, which was consistent with previous study [50]. The most interesting finding was pharmacists who often provided patients with breast cancer educational materials or self-assessment quizzes were more likely to have >50% knowledge score compared with those who rarely provide such service. Because, presence of educational materials in community pharmacies is beneficial to increase public awareness about breast cancer and support the pharmacist's role in breast cancer heath care promotion [28,55]. However, the contributions of pharmacist to promote public health issues are not widely reported, which may be partially due to some of their services goes unnoticed [64]. Furthermore, pharmacists spend much time to fulfill their responsibilities during working h such as dispensing, supplying, and management activities, and have no enough time for delivering professional pharmacy services [65]. More important, community pharmacists encounter numerous management challenges that would distract them from providing additional professional services [66].

In this study, community pharmacists showed positive attitude to be involved in breast cancer health promotion activities and to provide breast cancer educational materials in pharmacies [67]. While the key barrier that limits them to contribute in breast cancer education is lacks to get access to breast cancer educational materials as previously reported by community pharmacists in Qatar [49]. Therefore, cancer organizations should be encouraged to provide community pharmacies with printed educational materials pertained to breast cancer for public distribution [7,68]. This study indicated that pharmacists' knowledge of breast cancer and its screening methods are positively associated with the involving attitude in breast cancer promotion among pharmacists [69]. In this part, it is responsibility of pharmacists to take a pivotal role in breast cancer health education in community [31], as they have the potential to promote public health issues [64].

Jordan is considered a medium-income country with a population of young structure [70], and most of the patient are presented with progressive stages of breast cancer and more aggressive tumors type [71]. Accordingly, breast cancer adds more burden on the country's healthcare system. Therefore, much works and efforts should be focused on improving the access, awareness, and participation role of pharmacist in early detection to down-stage of breast cancer cases [7]. Jordan is considered the training center for healthcare professions in the region [70]. Nevertheless, much efforts are needed to improve breast cancer health promotion at both public and private levels and leaving pharmacy role with an incredible opportunity to extend. Accordingly, government and pharmacy governing bodies should work together to widen the scope of pharmacy practice and to extend the role of community pharmacists [72]. Additionally; pharmacists need to take responsibility for advocating the pharmacy as a profession and support the patient advocacy through build interactions and partnerships with patients for follow-up care [72]. Importantly, improving communication and social skills of community pharmacists with patients and other healthcare provider. This can be attained via universities which could offer a training course for students who are willing to work in community pharmacies. This would allow future students to achieve the practice of the profession in the community pharmacy in more efficient and appropriate way [63].

### Limitation of the study

The detailed information about breast cancer-specific education topics and training methods for the pharmacists was not obtained, as well as the patients and decision makers' opinions were not collected in this study. Therefore, further studies about breast cancer-specific education topics and on opinions of breast cancer patients and decision makers in health authorities in Jordan are recommended to mitigate existing misinterpretations and bridge knowledge gaps altogether.

## Conclusion

The present study indicates significant implications for pharmacist's knowledge toward key issues of breast cancer health promotion in Jordan. The findings show that most pharmacists in Jordan are lack sufficient knowledge about breast cancer and lack the access to educational materials about breast cancer health promotion. Importantly, it highlights the positive preparedness of pharmacists in Jordan to educating themselves about breast cancer and support incorporation of the educational program in the curriculum of university pharmacy school.

Up to my knowledge, no studies have been set out to explore further gaps, improvements, and subtle changes over time of community pharmacists' knowledge, attitudes, and barriers toward health promotion of breast cancer in Jordan especially after COVID-19 pandemic, and their role in promoting the health of patients with breast cancer visiting the pharmacies.

## Recommendation

Future research are encouraged in Jordan to assess the views and knowledge of patients and different healthcare providers about breast cancer promotion. This has the potential to alleviate existing misunderstandings about promotion of breast cancer health key issues and bridge knowledge gaps from different healthcare providers and patients altogether.

**Summary points**

- The objective of this study was to assess pharmacists' knowledge, attitudes, and barriers toward breast cancer health promotion among community pharmacists.
- An internet-based self-administrated questionnaire was rolled out using social media groups to the community pharmacists in Jordan.
- A 76.7% of the pharmacists had insufficient knowledge score of breast cancer and 92.7% had positive attitude. Access to breast cancer educational materials was the major barrier to pharmacists. A significant association was found between pharmacists' knowledge and breast cancer educational materials being given to patients (p < 0.001).
- This research detects some predictors for breast cancer knowledge amid contributed pharmacists.
- This study identifies the significance to initiate educational courses and to improve the access to educational material for community pharmacists about breast cancer health to increase public awareness to health services provided by pharmacists.
- Despite the low breast cancer knowledge score and stated barriers that could prevent actualizing community pharmacists' role, they had positive attitude toward educating patients about breast cancer health.
- The outcomes of this research support the essential for conducting further studies on identifying the approaches to integrating community pharmacists in the team of healthcare provider for breast cancer patients, overcoming all stated barriers, and to evaluate the knowledge and views of different healthcare providers and patients about breast cancer promotion are encouraged to improve the positive promotion and awareness of healthcare of breast cancer.
